# Inherited transthyretin cardiac amyloidosis presenting with diastolic heart failure and gastrointestinal symptoms: a case report and literature review

**DOI:** 10.3389/fcvm.2025.1588291

**Published:** 2025-05-08

**Authors:** Juan Wang, Run Zhang, Zhengliang Li, Wenzhong Zhang

**Affiliations:** The Affiliated Hospital of Qingdao University, Department of Cardiology, Qingdao, Shandong, China

**Keywords:** inherited transthyretin cardiac amyloidosis, echocardiography, cardiac magnetic resonance, technetium-99m pyrophosphate imaging, bone scintigraphy

## Abstract

The prevalence of Inherited transthyretin cardiac amyloidosis (hATTR-CA) is rising with an aging population and more mutation carriers. Its symptoms often resemble other heart diseases, leading to delayed diagnosis and affecting prognosis. With the advancement of noninvasive diagnostic methods, early detection and targeted treatment of hATTR-CA are becoming possible. However, better clinical awareness and diagnostic capabilities are still needed. This article reports a typical case of a 70-year-old man diagnosed with hATTR-CA. The patient presented with cardiac symptoms, including chronic chest tightness, breathlessness, and hypotension, as well as extracardiac symptoms such as chronic diarrhea. He also experienced two episodes of syncope, with symptoms progressively worsening. The diagnosis of hATTR-CA was confirmed following a comprehensive diagnostic work-up, including ECG, ambulatory blood pressure monitoring, echocardiography, cardiac magnetic resonance (CMR), bone scintigraphy, and genetic testing, in accordance with current guidelines. The patient has been treated with tafamidis, a transthyretin (TTR) tetramer stabilizer, for 3 months. While there was no significant improvement in ECG and echocardiography, the patient reported a marked reduction in chest tightness, breathlessness, hypotension, and gastrointestinal symptoms compared to before treatment. This case, along with a literature review, explores the clinical features, diagnostic methods, and treatment strategies of the disease, highlighting the importance of early diagnosis for prognosis, particularly the role of imaging in diagnosis.

## 1 Introduction

Inherited transthyretin cardiac amyloidosis (hATTR-CA) is a rare autosomal dominantly inherited disorder caused by TTR gene mutations (1). This leads to the deposition of transthyretin protein in the heart, leading to a restrictive pathology and resulting in diastolic dysfunction. This extracellular amyloid deposition disrupts the tissue structure mechanically and induces proteotoxicity, inflammation, generation of reactive oxygen species, apoptosis, and autophagy (2).

The disease has insidious symptoms and low detection rates, many cardiac and extracardiac factors should prompt suspicion of this disease. Patients are often diagnosed late, leading to refractory heart failure and a poor prognosis. Since 2016, when Gillmore and his colleagues introduced the non-invasive diagnostic algorithm, an authentic exponential increase in the number of diagnoses of transthyretin (TTR)-related CA was witnessed (3). As understanding of the disease has advanced, innovative therapies—from organ transplantation to transthyretin stabilizers (diflunisal, tafamidis, AG-1), TTR silencers [ALN-ATTR02, ISIS-TTR(Rx)], and degraders of amyloid fibrils (doxycycline/TUDCA)—have emerged, offering new potential and significantly improving treatment outcomes for ATTR-CA (4). With early diagnosis and active treatment, the patient's prognosis improved, symptoms of heart failure were controlled, and quality of life significantly improved.

This case report describes a 70-year-old male patient with chronic chest tightness, shortness of breath, low blood pressure, and chronic diarrhea. After tests including ECG, blood pressure monitoring, echocardiography, CMR, bone imaging, and genetic testing, he was diagnosed with hATTR-CA. Although imaging showed no significant improvement, the patient's symptoms improved significantly after treatment with the TTR stabilizer (tafamidis). This case emphasizes the importance of early diagnosis and accurate treatment of hATTR-CA, particularly the role of imaging in diagnosing myocardial infiltration.

## 2 Case presentation

A 70-year-old male with a history of hypertension and coronary artery disease came to our hospital with heart failure and gastrointestinal symptoms. Over the past six months, he experienced chest tightness, shortness of breath, palpitations, low blood pressure, diarrhea, and abdominal pain. His symptoms worsened in the last 10 days, with dizziness and syncope. He had been treated at local hospitals, where his ECG showed premature beats and ST-T changes, and echocardiography revealed enlarged atria, thickened ventricular walls, and reduced diastolic function. Cardiac CTA showed no significant coronary stenosis. As his symptoms persisted, he was referred to our hospital.

## 3 Diagnostic procedure

The patient was admitted with symptoms similar to previous hospitalizations. Physical examination showed normal respiratory rate, clear breath sounds in both lungs, and no rales (dry or wet). Cardiovascular examination revealed a regular heart rhythm with no obvious murmurs in the valve auscultation areas. Abdominal examination was soft with no rebound tenderness or masses. However, the patient had developed pitting edema in both lower limbs, which raised concern for potential circulatory system abnormalities.

After the initial assessment, based on the patient's symptoms and signs, we decided to further refine the diagnosis through a series of laboratory and imaging tests. A detailed summary of the tests conducted and their key results is provided in Table 1.

**Table 1:** Patient's laboratory and imaging findings.

Project name	Result	Abnormality	Unit	Reference rang
Laboratory markers				
Hb	129	↓	g/L	130–175
PT	10.9		sec	9.4–12.5
LDL-C	2.26		mmol/L	2.07–3.37
FPG	4.81		mmol/L	3.90–6.10
K	4.58		mmol/L	3.5–5.3
ALT	18		U/L	9–50
eGFR	89.21		ml/min/1.73 m^2^	
NT-proBNP	1,486.00	↑	pg/ml	0.00–125.00
Myoglobin	24.34	↓	ug/L	28.00–72.00
Hs-cTn	0.041	↑	ug/L	<0.014
CK	42	↓	U/L	50–310
CK-MB	10		U/L	0–24
Immunoelectrophoresis	Negative			
Immunoglobulin Light Chain	Negative			
Urinary *κ*-light chain	36.20	↑	mg/L	0–7.1
Urinary *λ*-light chain	7.19	↑	mg/L	0–3.9
Serum Free *κ*-Light Chain	32.87	↑	mg/L	3.30–19.40
Urinary Free *λ*-Light Chain	17.12	↑	mg/L	0.27–15.21
Electrophysiological study				
ECG	first-degree atrioventricular block, left anterior fascicular block, prolonged QTc interval, mild ST segment depression, non-specific ST-T changes			
Holter	sinus arrest (PR interval up to 2,234 ms), frequent atrial and ventricular premature beats, first-degree atrioventricular block, ventricular escape beats, non-specific intraventricular block, poor R-wave progression in V1-V3 leads, and dynamic ST-T changes			
ABPM	a mean blood pressure of 102/63 mmHg and a mean heart rate of 69 beats/minute			
Imagine				
Echocardiography	interventricular septum and left ventricular free wall thickness >12 mm, up to 17 mm; left ventricular ejection fraction (LVEF) 59%; E/A ratio 1.1, E/e' ratio 30.3; strain <−15%.			
MR	Bilateral atrial enlargement: LAD 78 mm, RAD 52 mm; LVEDd 52 mm, RVEDd 40 mm; LVEDV 212 ml, LVESV 112 ml; Reduced LV diastolic wall motion; Delayed enhancement in RV, LA, and IAS; Slightly reduced LV function: LVEF 47%; Increased T1 mapping: 1,474 ms; Increased ECV: 50%; Increased LV mass: 203 g; CO: 6.69 L/min.			
99mTc-PYP imaging	Significant increase in myocardial 99mTc-PYP uptake.			

Given the patient's history of chronic diarrhea accompanied by dizziness, we suspected the presence of hypotension, particularly due to dehydration that might have been caused by prolonged diarrhea. Therefore, we conducted a 24 h ambulatory blood pressure monitoring to comprehensively assess the patient's blood pressure fluctuations and further rule out hypotension.

At the same time, the patient's dizziness, along with episodes of syncope, raised concerns about potential abnormalities in the cardiac conduction system. Considering that these symptoms could be related to conduction block, we decided to arrange a 24 h dynamic electrocardiogram(ECG) to monitor heart rhythm changes and exclude potential arrhythmias or conduction issues.

The patient had chest tightness, shortness of breath, and syncope. Lab tests showed elevated high-sensitivity troponin, and the ECG showed poor R-wave progression in leads V1–V3, suggesting possible myocardial ischemia. However, coronary CTA showed only mild stenosis and no clear signs of ischemia. Therefore, we decided to perform an echocardiogram to rule out other cardiac issues, such as myocardial dysfunction, valve disease, or pericardial conditions.

The echocardiogram revealed abnormalities in the patient's heart structure and function, consistent with characteristics of CA. This finding raised a high suspicion of CA, particularly given that CA often leads to gradual heart stiffness and decreased function. However, its symptoms are similar to those of other heart diseases, making it prone to misdiagnosis. Although echocardiography plays an important role in screening for CA, it cannot determine the specific type of CA. Definitive diagnosis still requires additional diagnostic methods, including CMR, 99mTc-PYP scintigraphy, and genetic testing (5).

To confirm the type of amyloidosis, we performed CMR, which further validated the presence of myocardial amyloidosis and helped exclude other heart conditions. The patient's T1 mapping value of 1,474 ms and ECV of 50% suggest significant myocardial fibrosis or amyloid deposition, which is consistent with restrictive cardiomyopathy. These findings indicate the potential presence of cardiac amyloidosis and support further investigation into this diagnosis. However, it could not distinguish between ATTR-CA and other forms of amyloidosis.

The patient's laboratory results did not completely rule out other types of amyloidosis, particularly AL amyloidosis (light-chain type). Urinary protein immunofixation electrophoresis was slightly elevated, along with an increase in serum Kappa light chains and urinary Lambda light chains, which prompted us to consider the possibility of AL amyloidosis. According to current guidelines, AL amyloidosis cannot be excluded, as serum and urinary immunofixation combined with serum free light chain testing has a sensitivity of over 95% for detecting AL amyloidosis (6). However, after reviewing the literature, we found that diagnosing AL amyloidosis is complex because it may appear in up to 5% of the general population, with the prevalence increasing with age. It is also reported in 40%–50% of ATTRwt and ATTRVal122Ile patients (7). Therefore, although the laboratory results showed an increase in light chains, the increase was minimal, and the hematology experts considered it a trivial, non-significant elevation, which did not support the diagnosis of AL amyloidosis.

To address this uncertainty, we performed 99mTc-PYP scintigraphy, a nuclear imaging technique specifically used for diagnosing ATTR-CA. Previous studies have shown that 99mTc-PYP cardiac imaging has 97% sensitivity and 100% specificity in identifying ATTR-CA and can distinguish between ATTR and AL cardiac amyloidosis (8). The imaging results showed typical transthyretin deposition, confirming the diagnosis of ATTR-CA and effectively ruling out AL amyloidosis.

**Figure 1:**
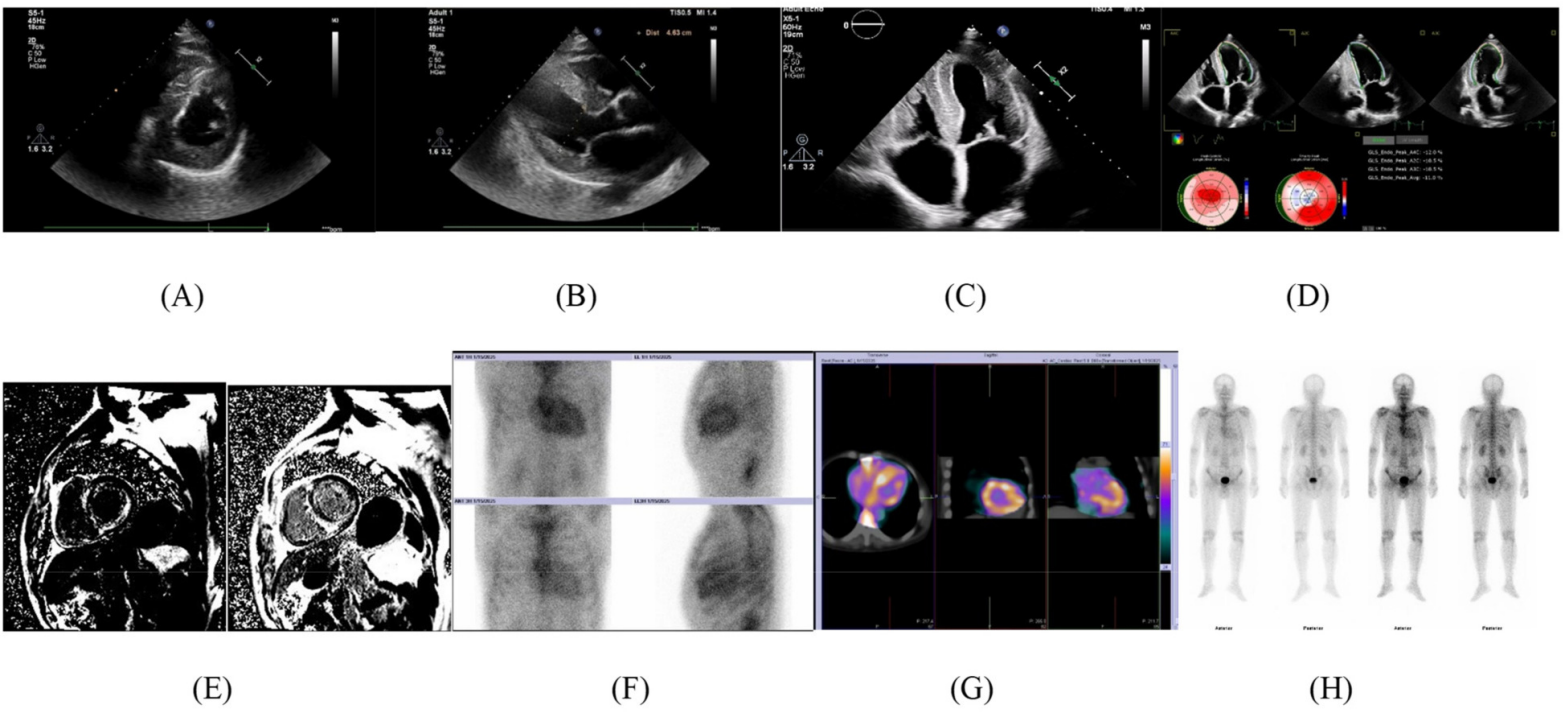
Imaging findings. **(A)** Fine punctate or granular echo enhancement in the interventricular septum. **(B)** Uniformly thickened myocardium with a ground-glass appearance. **(C)** Bilateral atrial enlargement. **(D)** Left ventricular longitudinal strain bullseye pattern/apical sparing phenomenon/strawberry sign. **(E)** MR: delayed enhancement shows “dust-like” enhancement in the left ventricle, right ventricle, left atrium, and interatrial septum. **(F)** ① Heart anterior and lateral views at 1 h and 3 h: The cardiac radiopharmaceutical shows diffuse increased uptake, while the ribs are poorly visible; ② Heart-to-lung ratio (H/CL) for the contralateral lung: 1 h: 1.77, 3 h: 1.51 (1 h H/CL > 1.5 is considered positive, 3 h H/CL > 1.3 is considered positive). ③ Visual score: 3 points (myocardial uptake is greater than rib uptake, with mild or no rib uptake). **(G)** SPECT/CT fusion imaging: Diffuse radiopharmaceutical uptake is mainly in the ventricular walls. **(H)** Whole-body imaging: Increased uptake in the heart, spine, kidneys, and bladder.

Genetic testing was conducted to differentiate between hereditary and wild-type ATTR-CA, confirming a TTR gene mutation and establishing a diagnosis of hereditary ATTR-CA. This condition is inherited in an autosomal dominant pattern, meaning there is a 50% chance of passing it on to offspring. The diagnosis has significant implications for the patient's family. With this confirmation, the diagnostic process is complete, and the disease's red flags and imaging findings are summarized in Table 2 and Figure 1.

**Table 2:** Red flags of imaging.

Red flags	
Symptoms	Multiple system involvement: 1. amyloidosis affects cardiac function leading to heart failure; 2. gastrointestinal involvement results in symptoms such as diarrhea and abdominal pain
ECG	1. conduction system disorder; 2. ischemic changes
Echocardiography	1. fine punctate or granular echo enhancement in the interventricular septum; 2. uniformly thickened myocardium with a ground-glass appearance; 3. restricted ventricular relaxation function; 4. bilateral atrial enlargement; 5. left ventricular longitudinal strain bullseye pattern/apical sparing phenomenon/strawberry sign; 6. small amount of pericardial effusion
MR	Left ventricular wall thickening, especially the interventricular septum, with reduced diastolic function. The interatrial septum is also thickened. Delayed enhancement shows “dust-like” enhancement in the left ventricle, right ventricle, left atrium, and interatrial septum.
99mTc-PYP imaging	Significant increase in myocardial wall 99mTc-PYP uptake

## 4 Treatment and prognosis

Upon confirmation, we immediately started treatment with Tafamidis, a medication that stabilizes transthyretin molecules and slows the progression of amyloidosis.

After three months of treatment, follow-up visits showed significant improvement in the patient's cardiac function, with a marked reduction in symptoms such as chest tightness and shortness of breath, leading to a substantial improvement in quality of life. In addition, we recommended that the patient undergo detailed family screening to identify potential genetic carriers and provide early interventions and preventive measures for their family members.

## 5 Discussion

CA is a progressive and potentially fatal heart disease caused by the deposition of abnormal proteins, leading to impaired heart function (9). Due to its symptoms being similar to other heart diseases, many patients are not diagnosed early, missing the optimal treatment window and increasing the risk of death. Timely identification of CA allows for effective treatment, improving prognosis (2).

### 5.1 Clinical feature

#### 5.1.1 Cardiac manifestations

Cardiac amyloidosis (CA) is an under-recognized cause of heart failure. Its early symptoms, such as heart failure and arrhythmias, are non-specific and often mistaken for other heart conditions, leading to delays in diagnosis and treatment.

##### 5.1.1.1 Valve diseases

Studies have found that this progressive infiltrative disease also affects the heart valves, with a particularly high prevalence of aortic valve stenosis (AS). CA and AS can coexist in elderly patients (>65 years), with approximately 4%–16% of AS cases presenting with ATTR-CA, especially in those undergoing transcatheter aortic valve replacement (TAVR) (10). In cases of severe stenosis, valve replacement remains the best option for achieving favorable outcomes. Further research is needed to determine the prognosis and optimal treatment strategies for patients with both CA and AS. In summary, the coexistence of CA and AS in elderly patients presents unique challenges, emphasizing the need for careful management and tailored treatment approaches.

##### 5.1.1.2 Arrhythmia

Cardiac amyloidosis is caused by amyloid protein deposits in the heart, leading to inflammation, oxidative stress, and fibrosis in heart cells. This can result in conduction problems like bundle branch block, sinoatrial node disease, atrioventricular block, and ventricular arrhythmias. There are two main types of cardiac amyloidosis: light chain cardiac amyloidosis (AL-CA) and transthyretin cardiac amyloidosis (ATTR-CA, which can be hereditary or wild-type) (11). Atrial arrhythmias are more common in wild-type ATTR-CA than in hereditary ATTR-CA or AL-CA. In cases with severe conduction issues or refractory arrhythmias, atrioventricular node ablation combined with pacemaker implantation can be an effective treatment, especially when other treatments fail (12). In conclusion, the management of cardiac amyloidosis requires careful attention to arrhythmias and conduction disorders, with specialized treatment options for refractory cases.

#### 5.1.2 Extracardiac manifestation

Amyloidosis, as a systemic disease, usually affects multiple organs, presenting with various clinical manifestations. In most patients, cardiac involvement occurs later, often with extra-cardiac symptoms appearing first.

##### 5.1.2.1 Hepatic involvement

Liver involvement is common in AL amyloidosis, with up to 90% of autopsies showing amyloid deposits (13). However, symptoms like anorexia, weight loss, and fatigue are often seen in many conditions, and it may only present with hepatomegaly and elevated ALP, making it clinically less significant. Due to high rates of misdiagnosis, persistent clinical investigation is essential for accurate diagnosis and treatment.

##### 5.1.2.2 Gastrointestinal involvement

Similar to this case, when amyloidosis affects the digestive system, symptoms like abdominal pain and diarrhea can occur. This is seen in 1% of primary systemic amyloidosis cases (14). Delayed diagnosis worsens the prognosis. Studies suggest the gut-heart axis may play a role in the development of heart diseases like restrictive cardiomyopathy, offering new treatment possibilities (15).

##### 5.1.2.3 Neurological involvement

In both senile and hereditary amyloidosis, patients commonly experience neuropathies like carpal tunnel syndrome (CTS), tendon ruptures, and sensory-motor polyneuropathy (16). CTS often appears 5–10 years before CA symptoms, making it useful for early screening (17). Spinal stenosis or nerve root issues may offer early clues for diagnosing amyloidosis. Surgeons should consider biopsies during surgery to identify amyloidosis and aid in post-op screening (18).

### 5.2 Diagnostic methods

Advances in diagnosis and treatment have now transformed CA from a rare and incurable disease to a more prevalent and manageable one.

#### 5.2.1 Routine cardiac imaging

Non-invasive diagnostic tools such as electrocardiograms, echocardiograms, and cardiac MRI can raise suspicion for CA; 99mTc-PYP scintigraphy can non-invasively confirm ATTR-CA.

##### 5.2.1.1 Electrocardiography

Electrocardiography (ECG) has diagnostic value in the identification of cardiac amyloidosis (CA). Common ECG features include low to normal QRS wave amplitude and various arrhythmias, such as atrial fibrillation and conduction blocks. These changes reflect alterations in myocardial structure and disruptions in electrophysiological function. However, the specificity of ECG is low, and its findings can overlap with those of other cardiac conditions, potentially leading to misdiagnosis or missed diagnosis. Therefore, ECG serves only as an early indicator, and a definitive diagnosis requires further imaging and laboratory tests.

##### 5.2.1.2 Echocardiography

An echocardiogram is performed and must assess left ventricular wall thickness, myocardial echo, atrial size (and function), septal and valve thickness, pericardial effusion, diastolic function including mitral inflow E and A wave ratios and deceleration times, tissue Doppler s', e', and a' velocities, and estimation of pulmonary artery systolic pressure and right atrial pressure. Characterisation of longitudinal left ventricular strain using speckle tracking echocardiography, including the bull's eye view, is also recommended (19). Despite the value of echocardiography in screening for CA, it does not confirm the type of CA and a definitive diagnosis still requires a combination of other ancillary tests such as cardiac magnetic resonance, 99Tc m-PYP imaging and myocardial biopsy.

##### 5.2.1.3 Cardiac magnetic resonance

CMR plays a key role in the diagnosis of CA, alongside echocardiography. Characteristic CMR manifestations of CA include diffuse or transmural late gadolinium enhancement (LGE), abnormal gadolinium kinetics and high extracellular volume (ECV) (20).CMR LGE is more sensitive than echocardiography in detecting cardiac amyloidosis and distinguishes it from other causes of cardiomyopathy, with a sensitivity of up to 88 per cent and specificity of up to 92 percent (21). Although echocardiography and CMR are excellent for diagnosing CA, the inability to differentiate between CA subtypes requires further evaluation before a definitive diagnosis can be made.

##### 5.2.1.4 99mTc-PYP scintigraphy

Recent advances in bone scintigraphy and specialised tracers have made this imaging modality a cornerstone of CA assessment. Bone scintigraphy with technetium 99m [99mTc pyrophosphate (PYP], 99mTc, 3,3-diphosphono-1,2-propionic dicarboxylic acid [DPD], or 99mTc hydroxymethylene diphosphonate [HMDP]] should be performed in all patients with suspected CA. Current clinical practice supports the use of 99mTc derivatives to differentiate between ATTR and AL amyloidosis. 99mTc-PYP highly specific and highly predictive of ATTR disease, avoids the need for endocardial myocardial biopsy (19).

#### 5.2.2 Artificial intelligence (AI)

As awareness of CA has improved, so have various diagnostic modalities. Studies have shown that AI-enhanced ECG models are effective in detecting CA and may provide a valuable tool for early detection and intervention of this disease (22). Cardiac magnetic resonance offers unique imaging biomarkers as surrogate endpoints for clinical trials, with LGE and T1 labelling for replacement and interstitial fibrosis, T2-weighted imaging and T2 labelling for intramyocardial oedema, and T2* for iron contributing to unparalleled quantitative tissue characterisation, but their clinical significance needs to be continually assessed (23). Quantitative techniques that can be facilitated by AI in 99mTc-PYP studies are expected to improve diagnostic accuracy and risk assessment, and future research should focus on elucidating the clinical role of quantification and the best methods for doing so (24).

### 5.3 Treatment programmes

#### 5.3.1 Historical treatments

Historically, conventional treatments have been primarily supportive, with the primary aim of relieving symptoms, including treatments for heart failure symptoms, arrhythmias and conduction disturbances. For heart failure, dietary counselling, sodium (salt) intake restriction and weight control are provided. For arrhythmias, oral anticoagulants and amiodarone are often given directly (25). Permanent pacemaker implantation and cardiac resynchronisation therapy are the most common treatments for conduction disorders. Unconventional treatment is autologous peripheral blood stem cell transplantation (ASCT) after high-dose treatment with melphalan for AL-CA and liver and heart transplantation for ATTR-CA.

#### 5.3.2 Current novelty approaches

The newest form of monoclonal antibody targeting is plasma cells. A human monoclonal antibody called daratumumab, which targets CD38, is significantly expressed in abnormal plasma cells. Daratumumab shows promising results in early study in patients with AL amyloidosis (26). In addition to liver transplantation, the clinical efficacy of transthyretin (TTR) tetramer stabilisers and TTR gene silencers in hereditary ATTR (ATTRv) amyloidosis has been established (27). New drugs targeting ATTR-type myocardial amyloidosis (tafamidis, diflunisal) have been approved and shown to slow disease progression.

Due to the diverse pathogenesis of CA, it often presents with multiple clinical features, making it difficult to diagnose. This is further complicated by its similarities to more common diseases. However, thanks to advances in diagnostic techniques, pharmacological treatments and artificial intelligence, it is now possible to make an early diagnosis and start treatment immediately. And all diagnostic methods should be used together to ensure the most accurate diagnosis.

## 6 Conclusion

This case report underscores the importance of an integrated diagnostic approach, particularly the use of multimodal imaging, in the early identification of ATTR-type myocardial amyloidosis. By leveraging multidisciplinary collaboration and advanced diagnostic tools, timely recognition and appropriate treatment can be initiated, significantly improving the patient's quality of life. As genetic testing advances and new therapies emerge, the outlook for treating ATTR-type myocardial amyloidosis will continue to improve.

## Data Availability

The datasets presented in this study can be found in online repositories. The names of the repository/repositories and accession number(s) can be found in the article/Supplementary Material.
