# Supplementary figures and images for: Inherited transthyretin cardiac amyloidosis presenting with diastolic heart failure and gastrointestinal symptoms: a case report and literature review
Source: Front Cardiovasc Med. 2025 May 8;12:1588291. doi: 10.3389/fcvm.2025.1588291 (PMC12095264; doi:10.3389/fcvm.2025.1588291)

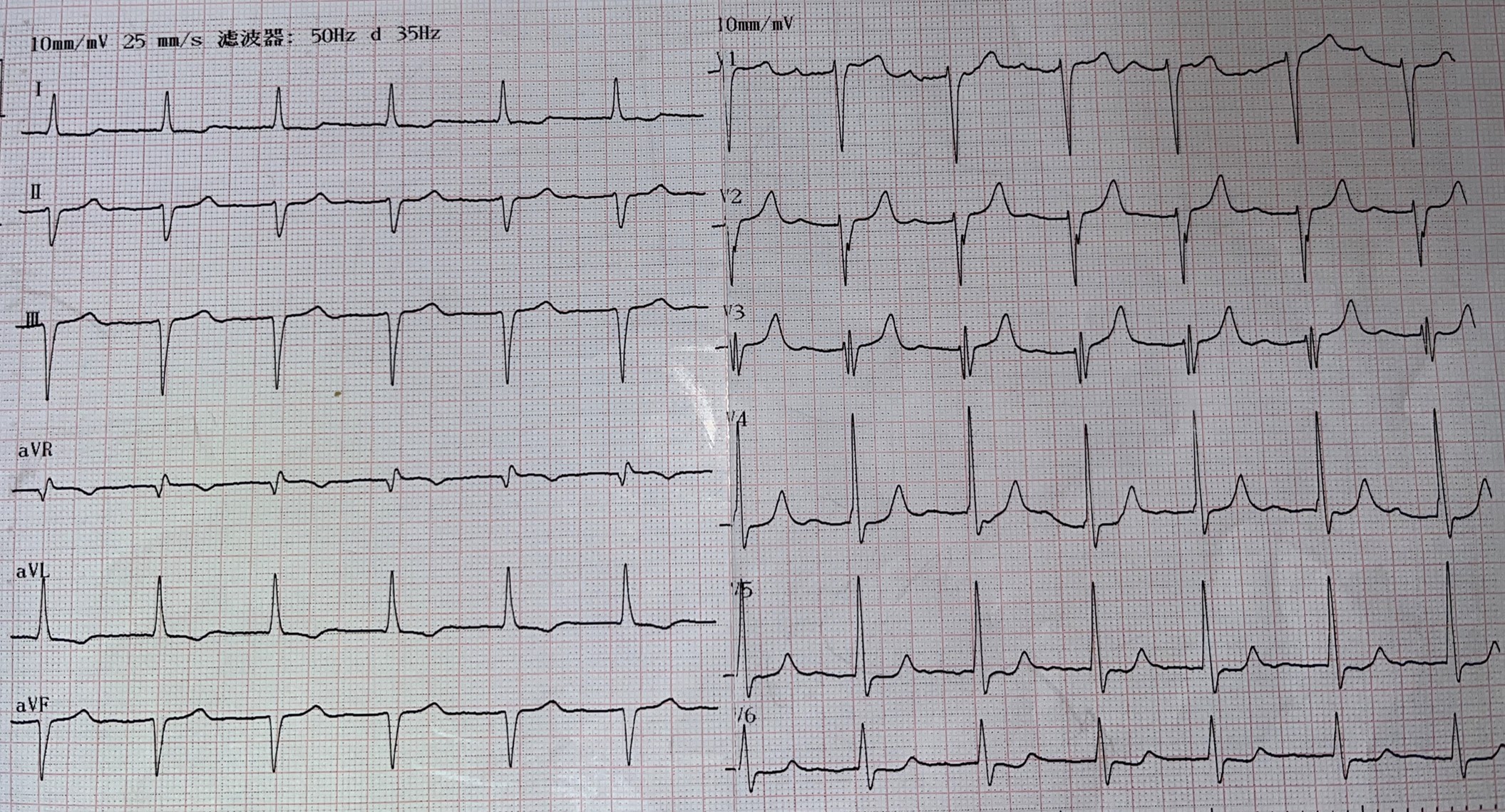

Supplement: Supplementary file 1 [file Datasheet1.zip › Supplementary Material/Figure 1. ECG.jpg]

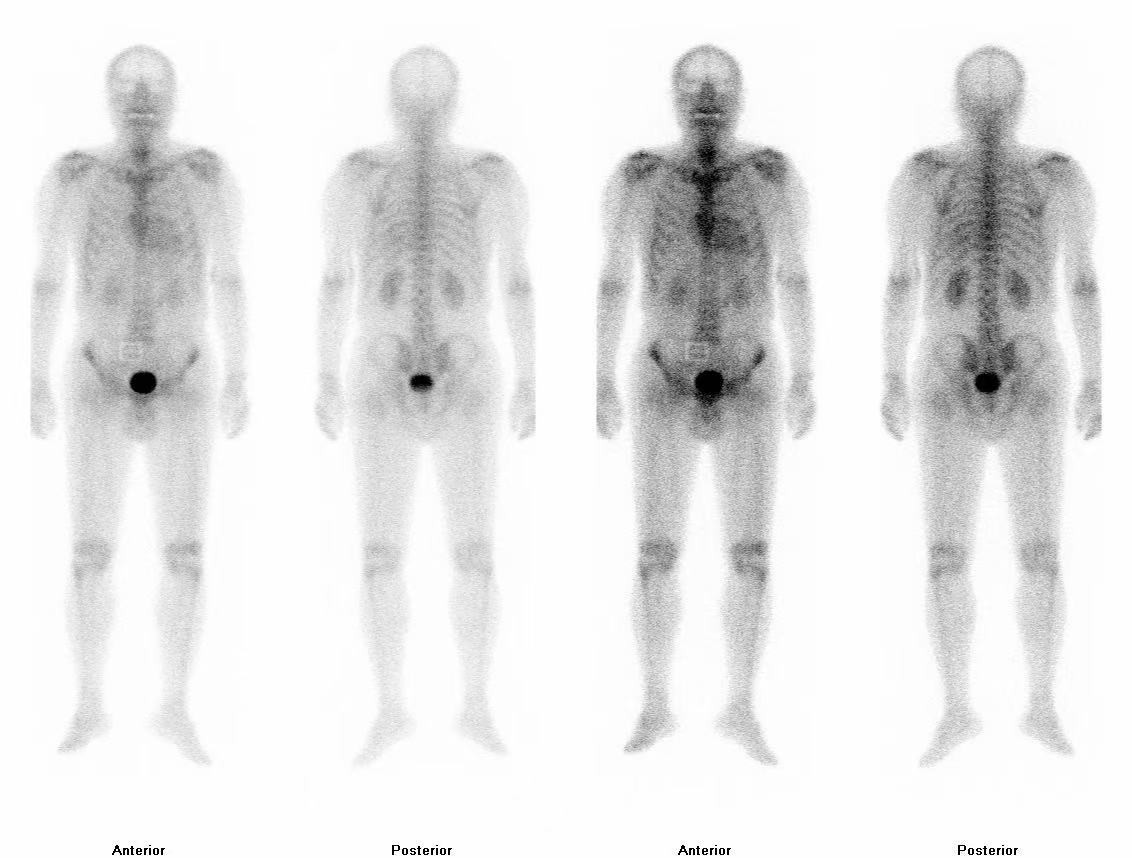

Supplement: Supplementary file 1 [file Datasheet1.zip › Supplementary Material/Figure 10. Whole Body Imaging:Cardiac uptake is increased, showing the midbone, both kidneys and bladder..jpg]

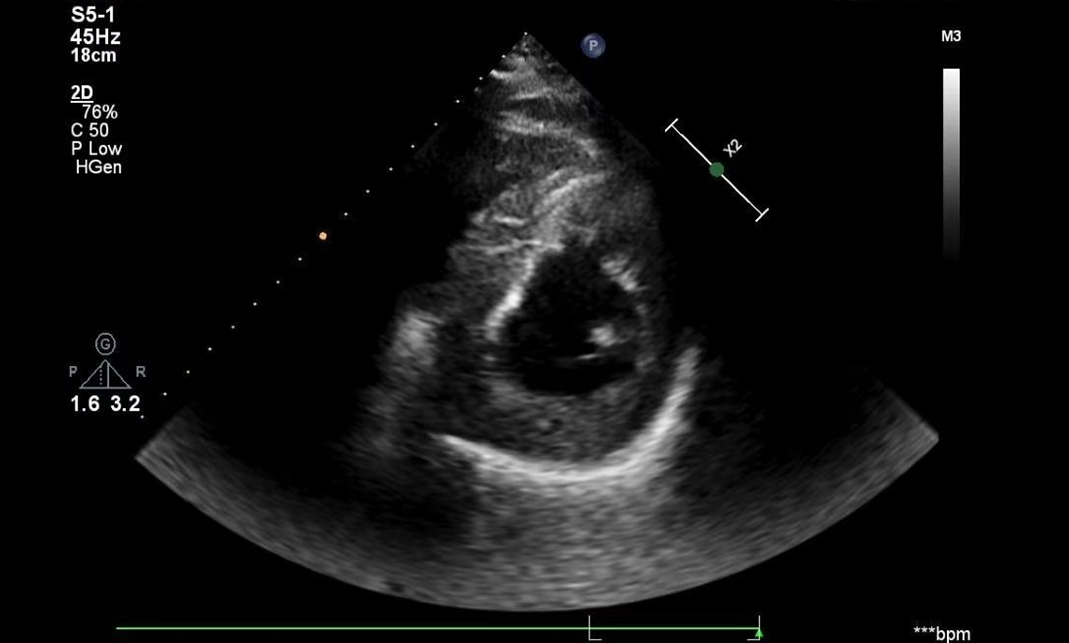

Supplement: Supplementary file 1 [file Datasheet1.zip › Supplementary Material/Figure 2. Fine dot-like enhancement on myocardial echoes of the ventricular septum.png]

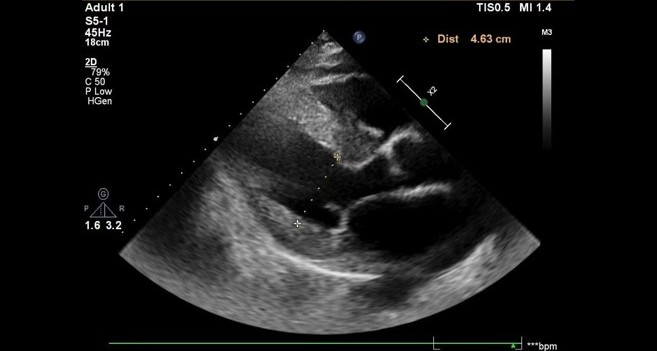

Supplement: Supplementary file 1 [file Datasheet1.zip › Supplementary Material/Figure 3. Homogeneous hypertrophic myocardium of the interventricular septum and left ventricular free wall with ground-glass alterations.jpg]

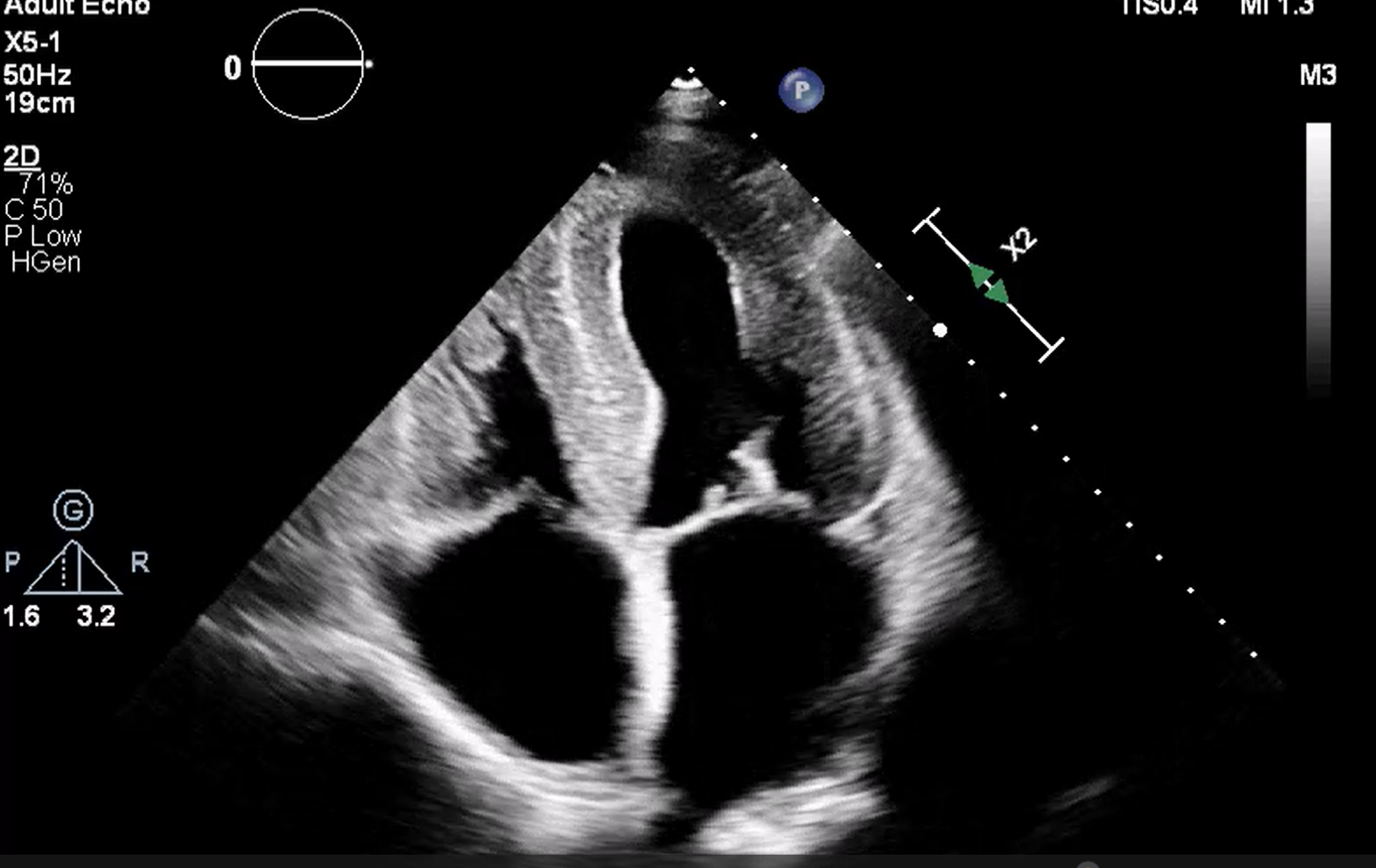

Supplement: Supplementary file 1 [file Datasheet1.zip › Supplementary Material/Figure 4. Enlargement of both atria.png]

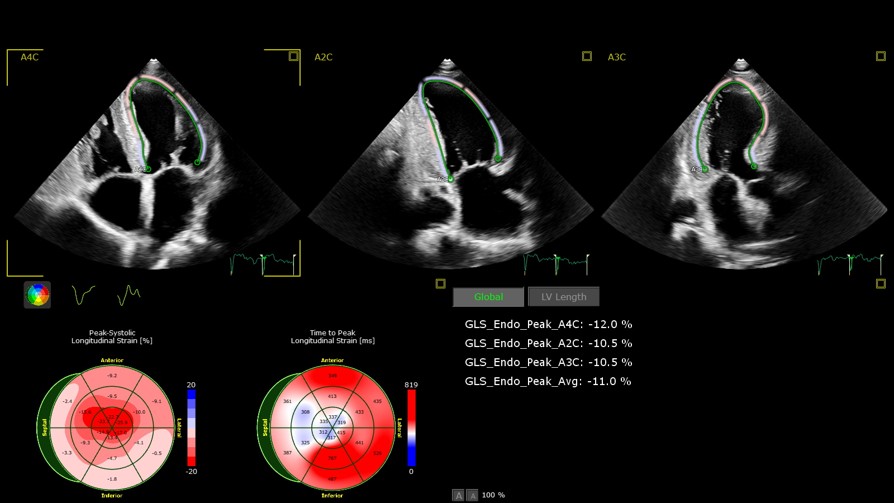

Supplement: Supplementary file 1 [file Datasheet1.zip › Supplementary Material/Figure 5. Longitudinal left ventricular strain indicating apical retention, also known as the “strawberry sign” or “bull's-eye view”.jpg]

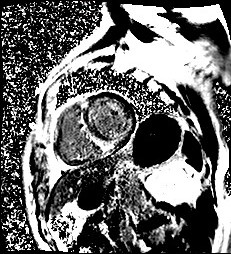

Supplement: Supplementary file 1 [file Datasheet1.zip › Supplementary Material/Figure 6. extensive subendocardial transmural 'dust' enhancement of the left ventricle, right ventricle, left atrium and interventricular septum (2).jpg]

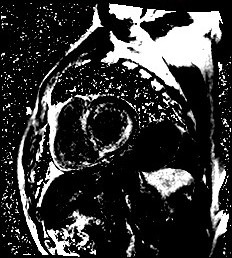

Supplement: Supplementary file 1 [file Datasheet1.zip › Supplementary Material/Figure 7. extensive subendocardial transmural 'dust' enhancement of the left ventricle, right ventricle, left atrium and interventricular septum (3).jpg]

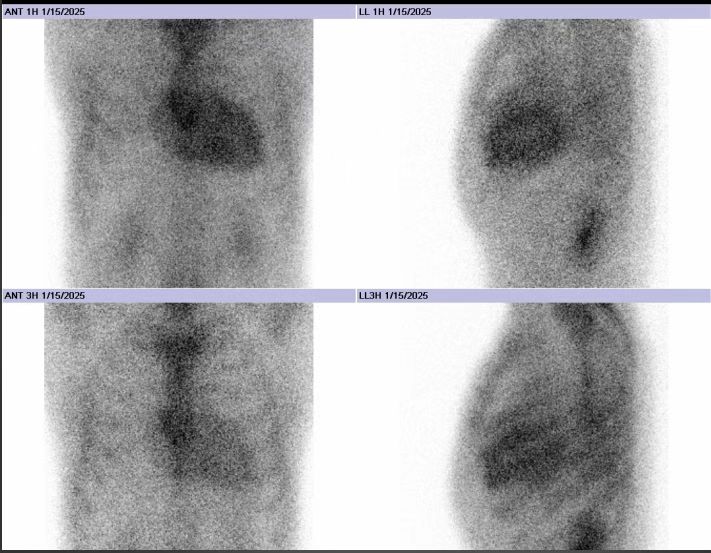

Supplement: Supplementary file 1 [file Datasheet1.zip › Supplementary Material/Figure 8..jpg]

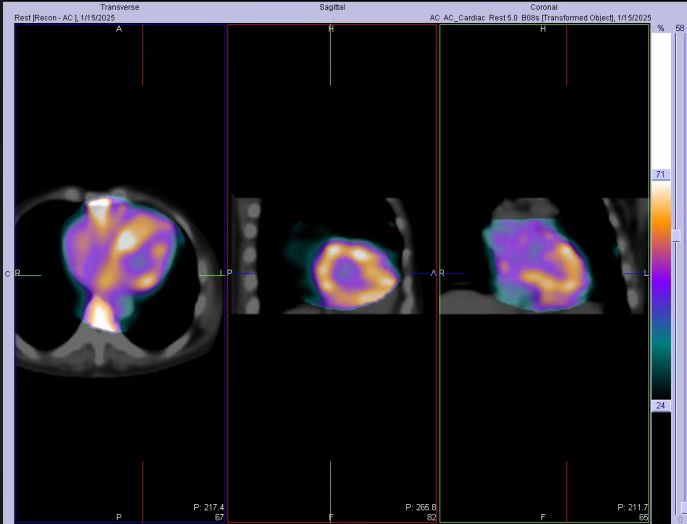

Supplement: Supplementary file 1 [file Datasheet1.zip › Supplementary Material/Figure 9. Tomosynthesis SPECTCT:Increased diffuse cardiac uptake predominantly in the ventricular wall..jpg]

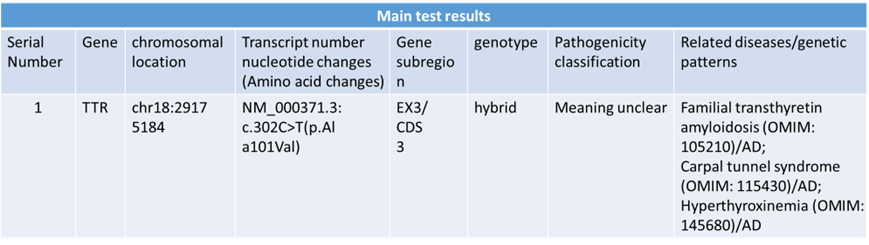

Supplement: Supplementary file 1 [file Datasheet1.zip › Supplementary Material/Table 1. Genetic testing results.png]
