# Supplementary material for: Inherited transthyretin cardiac amyloidosis presenting with diastolic heart failure and gastrointestinal symptoms: a case report and literature review
Source: Front Cardiovasc Med. 2025 May 8;12:1588291. doi: 10.3389/fcvm.2025.1588291 (PMC12095264; doi:10.3389/fcvm.2025.1588291)
Supplement: Supplementary file 1 [file Datasheet1.zip › Supplementary Material/Description of the Supplementary Material.docx]

Dear Editor and Reviewers:

Thank you for reviewing our manuscript. Below is a detailed explanation of the supplementary materials, including the interpretation of the figures and imaging data provided.

**Figure 1**: This figure displays the patient's electrocardiogram (ECG) upon admission, highlighting typical electrocardiographic abnormalities associated with the patient's cardiac pathology. These abnormalities offer initial clues for further diagnosis, suggesting potential cardiac dysfunction.

**Figures 2 through 4 and Video 1**: These images from echocardiography provide crucial signs indicative of the risk of cardiac amyloidosis (CA):

Figure 2 shows fine granular echoes in the interventricular septum, a finding highly suggestive of myocardial amyloidosis and a key imaging feature in early-stage disease.

Figure 3 demonstrates homogeneous myocardial thickening with a ground-glass appearance, a typical manifestation of myocardial involvement in CA.

Figure 4 illustrates biatrial enlargement, commonly associated with the progressive cardiac dysfunction seen in amyloidosis.

Video 1 further reveals impaired diastolic function of the heart, which is a characteristic feature of cardiac amyloidosis, indicating a restriction in the heart's ability to fill during diastole.

**Figures 5 and 6**: These figures present cardiac magnetic resonance imaging (CMR) findings, specifically delayed enhancement. Delayed enhancement is a hallmark of CA, indicating myocardial fibrosis or amyloid deposition.

**Figures 8 through 10**: These images depict bone scintigraphy findings, which provide further support for the diagnosis of ATTR-CA (transthyretin-related cardiac amyloidosis). All images demonstrate significantly higher uptake of technetium in the myocardial region compared to other areas, a classic feature of cardiac amyloidosis.

**Table 1:** This table presents the genetic testing results, confirming that the patient carries a mutation in the TTR gene, which definitively diagnoses the patient with hereditary ATTR-CA (hATTR-CA). Genetic testing results complement the imaging findings and provide further confirmation of the diagnosis.

Together, these figures and diagnostic results provide a comprehensive view of the diagnostic process for hATTR-CA, demonstrating the critical role of imaging and genetic testing in the early identification of this disease.

Thank you again for your review. We look forward to your valuable feedback and suggestions.
